# Nutritional Knowledge, Attitudes, and Practices of Patients With Chronic Inflammatory Rheumatism: Insights From a Patient Survey

**DOI:** 10.7759/cureus.74388

**Published:** 2024-11-25

**Authors:** Imane Bensaghir, Hanan Rkain, Nada Benzine, Sara Farih, Naima Chakyr, Fatine Kronbi, Hind L'heri, Laila Najdi, Najia Hajjaj-Hassouni, Latifa Tahiri, Fadoua Allali

**Affiliations:** 1 Rheumatology, Ayachi Hospital, Ibn Sina Hospital Center, Mohammed V University, Rabat, MAR; 2 Exercise Physiology and Autonomous Nervous System, Mohammed V University, Rabat, MAR; 3 Patient Association, Moroccan Association of Polyarthritics and Spondyloarthritics (AMPS), Casablanca, MAR; 4 Medicine, International University of Rabat, Rabat, MAR

**Keywords:** ankylosing spondylitis, dairy products, diet, gluten, nutrition, red meats, rheumatoid arthritis

## Abstract

Introduction

The objectives of this study are to assess the knowledge, attitudes, and practices of patients with chronic inflammatory rheumatism (CIR) about diet. Aiming to identify their level of understanding of the role of nutrition in symptom management, explore their perceptions about different types of foods, and analyze their current dietary habits. The study also aims to assess the impact of this knowledge and dietary changes on their CIR management.

Material and methods

This is a descriptive and analytical study carried out by the Moroccan Association for Research and Assistance to Rheumatism (AMRAR) in collaboration with the Moroccan Association of Polyarthritics and Spondyloarthritics (AMPS). By telephone interviews, we collected patients' sociodemographic data, knowledge, attitudes, and practices toward diet, using a structured Google form questionnaire validated by a team of rheumatologists. A univariate and multivariate analysis was performed to assess the factors associated with these elements.

Results

Two hundred patients participated in the survey (mean age: 48.3 ± 14.2 years, 77% female, 24.5% illiterate). Patients were followed up for rheumatoid arthritis (RA), ankylosing spondylitis (AS), psoriatic arthritis (PsA), and non-specified rheumatism in 59.5%, 25.5%, 3%, and 12% of cases, respectively.

An assessment of the nutritional knowledge of patients with CIR indicated a general awareness of the role of diet in disease management. Valid response rates for key statements ranged from 53.5% to 79%. However, a substantial number of patients (up to 40.5%) exhibited misconceptions, most notably regarding the consumption of red meat and dairy products.

Patients have varying perceptions of the impact of foods on rheumatic symptoms. For example, 36.5% think cow's milk aggravates symptoms, while 40% do not know. White flour is perceived as aggravating by 51.5% of patients, and red meat by 49.5%. On the other hand, foods such as ginger (46.5%) and turmeric (46%) are considered beneficial for symptoms, as are fish, considered to improve symptoms by 55% of patients. Finally, white sugar, soft drinks, and deli meats are thought to aggravate symptoms by 48.5%, 47.5%, and 51.5% of patients, respectively.

Among patients with CIR, 38% have modified their eating habits, the most frequent changes being a reduction in sugar, fatty foods, and red meat consumption by 51.4%, 52.7% and 55.5% of patients, respectively. In terms of cooking methods, 79.5% of patients prefer to bake, and 29.5% follow a specific diet, mainly Mediterranean (53%). 3.5% of patients stopped their medication, and 12% reduced their dose after having adopted these dietary modifications.

Conclusion

This study reveals significant gaps in the nutritional knowledge of patients with CIR. The varied and often incorrect perceptions of the impact of food on symptoms show the need for better nutritional education. To enhance CIR management, it is essential to develop specific dietary guidelines tailored to the Moroccan context.

## Introduction

Food is an integral part of every individual's life, representing one of the links we have with the environment and our lifestyles, and is intimately linked to our state of health. Outside any pathological context, and within the framework of prevention policies, there are dietary and nutritional recommendations for the general health of Moroccans [1].

In the management of patients suffering from chronic inflammatory rheumatism (CIR), non-pharmacological measures remain essential, as shown by the recommendations of the French Society of Rheumatology (SFR) [2,3]. Together with medication, these measures help to control disease activity and improve overall patient management. Among non-pharmacological measures, diet could be of particular interest in modulating the immune and/or inflammatory response, and have an effect on cardiovascular risk [4]. In this respect, dietary measures were already mentioned in the recommendations for rheumatoid arthritis (RA) [2].

The aims of this work are to assess the knowledge, attitudes, and practices of patients with CIR about diet. This includes identifying their level of understanding of the role of diet in managing their symptoms, exploring their perceptions and motivations for dietary change, and analyzing their current dietary habits. The study also aims to assess the impact of this knowledge and practice on CIR management.

This article was previously presented as a poster at the annual congress of SFR on December 10, 2023.

## Materials and methods

Study design

This is a descriptive and analytical cross-sectional study, carried out between August 2023 and December 2023, including all patients followed up for CIR presenting to the rheumatology department of Ayachi University Hospital, Salé, Morocco. This study was carried out by the Moroccan Association for Research and Assistance to Rheumatism (AMRAR) in collaboration with the Moroccan Association of Polyarthritics and Spondyloarthritics (AMPS). This study was conducted in accordance with the ethical standards of the 1964 Declaration of Helsinki and was approved by the Ethics Committee for Biomedical Research of Mohammed V University in Rabat (147/24).

Population

In this survey, patients with CIR were recruited. Subjects were included if they met the American College of Rheumatology (ACR)/European Alliance of Associations for Rheumatology (EULAR) 2010 criteria for RA and the Assessment of Spondyloarthritis International Society (ASAS) criteria for SpA if they had been followed for more than six months for their rheumatic disease, and if they were over 18 years of age [5,6]. Patients with psychiatric disorders and those who refused to participate in the study were excluded. All patients gave their consent before taking part in the study.

Questionnaire

In this study, the assessment of patients' knowledge, attitudes, and practices toward diet was carried out using a questionnaire, which was developed by a team of rheumatologists. The questionnaire consisted of three parts. The first part of the questionnaire covered patients' socio-demographic data, such as age, gender, level of education, housing, occupation, monthly family income, social security coverage, type of rheumatism, duration of illness, co-morbidities, medication used, and tobacco use. The second part assessed patients' knowledge of diet in CIR, using closed questions (yes, no, and I don't know) to determine whether patients were aware of the role of diet in the management of their disease and associated comorbidities. The third part explored patients' attitudes to diet, including questions on their beliefs about the impact of 63 different foods and drinks (see list in Appendix section) and the impact of certain dietary practices, such as fasting, on their rheumatic symptoms. Finally, the fourth part looked at patients' dietary practices, examining the diets they follow, the use of food supplements, and changes in their eating habits in response to their illness (see questionnaire in Appendix section).

Statistical analysis

Data were collected and analyzed using IBM SPSS Statistics software, version 21.0 (version 2012; IBM Corp., Armonk, New York, USA). A descriptive analysis was performed, and categorical variables were expressed as numbers and percentages and quantitative variables as means and standard deviations or medians and quartiles, depending on the distribution of the variable. Next, a univariate and multivariate analysis was performed to investigate the association between sociodemographic and clinical factors and patients' beliefs about the impact of food on joint symptoms. Additionally, the analysis explored the factors influencing non-adherence to therapy.

## Results

Patient characteristics

Two hundred patients participated in the survey (mean age: 48.3 ± 14.2 years, 77% female, 24.5% illiterate). Patients were followed for RA, AS, psoriatic arthritis (PsA), and non-specified rheumatism in 59.5%, 25.5%, 3%, and 12% of cases, respectively. Table 1 shows socio-demographic characteristics and disease data, including type of rheumatism, median duration, and current treatments.

**Table 1:** Sociodemographic and clinical characteristics of patients *Data presented as mean ± SD **Data presented as a percentage (%) ***Data presented as median CIR, chronic inflammatory rheumatism; DMARD, disease-modifying anti-rheumatic drugs; TNFi, tumor necrosis factor-alpha inhibitors; RA, rheumatoid arthritis; AS, ankylosing spondylitis; PsA, psoriatic arthritis

Parameters	N=200	Frequency
Age (years ± SD)*	48.3 ± 14.2	
Gender (%)**		
Female	77	154
Male	23	46
Habitat (%)**		
Urban	88	176
Rural	12	24
Level of education (%)**		
Illiterate	24.5	49
Primary school	13	26
Secondary school	10.5	21
High school	16.5	33
University	35.5	71
Marital status (%)**		
Single	27	54
Married	57	114
Divorced	7	14
Widowed	9	18
Economic status (%)**		
Below minimum wage	59	118
Above minimum wage	41	82
Comorbidities (%)**		
Hypertension	20.5	41
Diabetes	14.5	29
Gastrointestinal disorders	4	8
Heart disease	6.5	13
CIR		
Disease duration (years)***	8 (4;15)	
Type of CIR (%)**		
RA	59.5	119
AS	25.5	51
PsA	3	6
Non-specified	12	24
Conventional DMARDs (%)**		
Methotrexate	46.5	93
Sulfasalazine	9.5	19
Leflunomide	10.5	21
Biological DMARDs: N=64**		
Rituximab (%)	34.4	31
TNFi (%)	56.8	25
Tocilizumab (%)	4.4	4
Secukinumab (%)	4.4	4

Patients' nutritional knowledge

In this study, we assessed the nutritional knowledge of patients with CIR, based on the latest recommendations of the SFR. Patients were asked a set of questions covering different aspects of nutrition relevant to their condition. Table 2 shows the distribution of responses obtained for each question. Responses were categorized into three groups: "valid answers," corresponding to knowledge aligned with SFR recommendations; "invalid answers," representing incorrect or erroneous knowledge; and "no answer" when patients did not provide a response.

**Table 2:** Patients' nutritional knowledge CIR, chronic inflammatory rheumatism

Statement	Valid answers (%)	Invalid answers (%)	No answer (%)
Diet plays an important role in CIR management	59	4.5	36.5
Diet plays an important role in the management of CIR comorbidities	61	1	38
There is a link between diet and CIR activity	53.5	4	42.5
Weight loss (if overweight) helps control CIR activity	79	1	20
Weight loss (if overweight) helps control CIR comorbidities	76.5	0.5	23
Dietary practices must take into account the patient's age	57.5	7.5	35
Dietary practices must take into account the patient's current medications	62	3	35
Dietary practices must take into account the patient's comorbidities	70.5	2	27.5
CIR patients should not take dairy products	13.5	37	49.5
CIR patients should not eat red meats	14.5	40.5	45
CIR patients should favor fruits	62	6	32
CIR patients should favor vegetables	72	1.5	26.5
CIR patients should favor fish	68.5	2.5	29
CIR patients should favor olive oil	69	2	29
A well-balanced diet could replace CIR drugs	47	12	41

Patients' attitudes toward nutrition

Beliefs About the Influence of Different Foods on Rheumatism Activity

**Figure 1:**
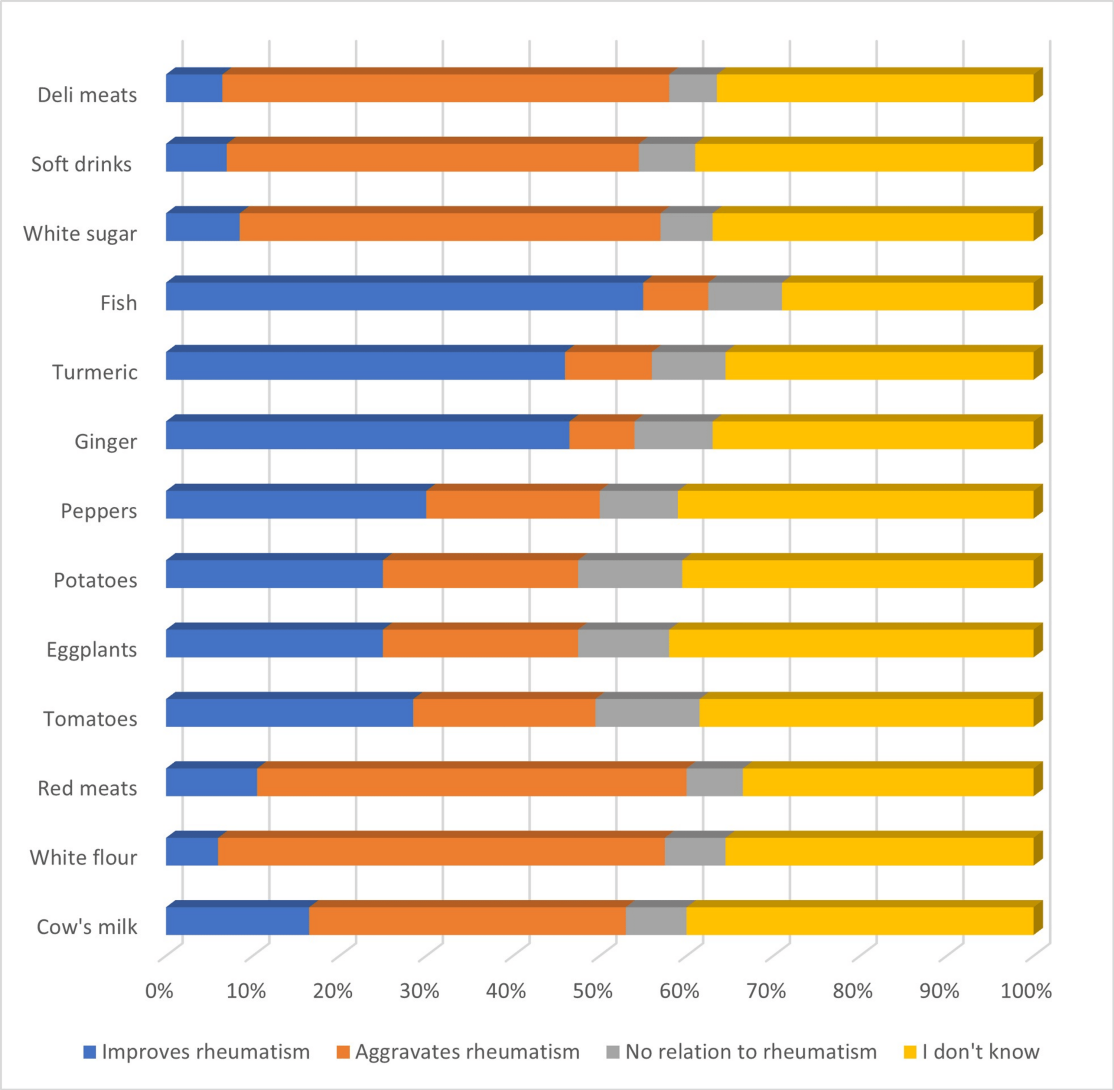
Patients' perceptions regarding the impact of food on rheumatism symptoms Data presented as a percentage (%)

We collected patients' perceptions regarding the effect of different foods on their joint symptoms (pain and stiffness). The data are expressed in percentages and fall into four categories: “Improves rheumatism,” “Aggravates rheumatism,” “No relation to rheumatism,” and “I don't know.” The foods studied were as follows: Cow's milk, white flour, red meats, Solanaceae (tomatoes, eggplants, potatoes, and peppers), ginger, turmeric, fish, white sugar, soft drinks, and deli meats (Figure 1).

Perceived Effects of Fasting and Dietary Modifications on CIR Symptoms

55% believe that fasting improves the symptoms of CIR. 64.6% of patients felt a difference in their overall health by changing their eating habits; improvements felt included weight loss (62.7%), increased energy levels (54.2%), better mobility (54.9%), reduced levels of pain (65.5%), reduced joint stiffness (43.7%), and improved overall mood (51.4%). Finally, with regard to the idea that a healthy diet could cure rheumatism without any need for medication, 8.5% of patients answered positively, 57% answered negatively, and 34.5% expressed uncertainty.

Patients' nutrition practices

Changes in Eating Habits

**Figure 2:**
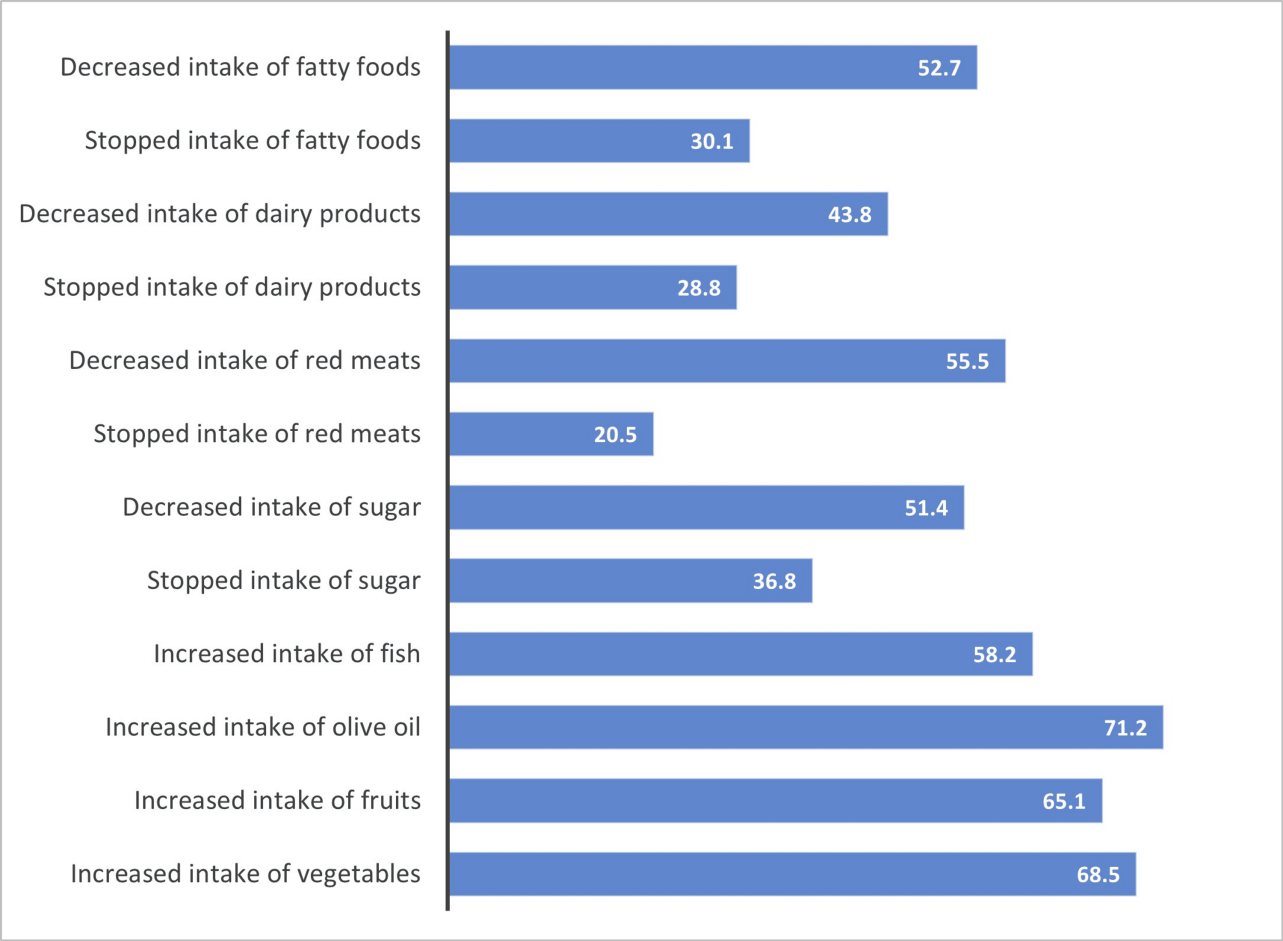
Dietary changes of CIR Data presented as a percentage (%). CIR, chronic inflammatory rheumatism

Since the diagnosis of their CIR, 38% said they had changed their eating habits. The changes made are shown in Figure 2.

Cooking Methods and Following a Special Diet

Patients were questioned about the cooking methods they adopt most often, 79.5% said they prefer baking, followed by 58.5% who opted for steaming and 58.5% for boiling food in water. Grilling was adopted by 45.5% of patients and around 38.5% chose to pan-fry their food, while 17.5% favored frying in oil as their main cooking method.

As far as diets are concerned, 29.5% said they were following a particular diet. The diets adopted included the Mediterranean diet (53%), sugar-free diet (46.2%), lactose-free diet (25.6%), the vegetarian diet (15.4%), gluten-free diet (10.3%), and the ketogenic diet (3.4%).

Dietary Supplements

31.5% of patients reported taking dietary supplements. The supplements most commonly taken by patients are vitamin D, consumed by 54.2% of respondents, followed by turmeric, taken by 39.8% of patients. Multivitamins, apart from vitamin D, were used by 34.9% of patients, and 31.3% took omega-3s. Herbal remedies were consumed by 21.7% of patients, while 15.7% took probiotics. Royal jelly was consumed by 14.5% of patients and spirulina (8.4%).

The Practice of Fasting

27% of patients practiced fasting to improve CIR symptoms, of whom 55% practiced intermittent fasting while 45% practiced dry fasting.

Stopping or Reducing the Dose of CIR Medication

Of the 200 patients surveyed, 3.5% said they had completely stopped their treatment after changing their dietary practices. However, 12% of patients claimed to have reduced the dose of their medication following dietary changes.

Univariate and multivariate analysis

Factors Associated With Patients' Beliefs About Diet

In univariate and multivariate analysis, the factor associated with the belief that cow's milk aggravates CIR symptoms was university education (OR: 0.108; 95% CI (0.038 - 0.301); p < 0.001); the results are shown in Table 3.

**Table 3:** Univariate and multivariate analysis of the belief that cow's milk aggravates CIR symptoms *P-value significant at <0.05. P, probability of error; RA, rheumatoid arthritis

Parameters	Univariate analysis		Multivariate analysis	
	OR (CI 95%)	P-value	OR (CI 95%)	P-value
Age	​​​​​​1.01 (0.010 - 0.030)	0.356	-	-
Gender	1.1 (0.592 - 0.786)	0.783	-	-
Level of institution				
Primary school vs illiterate	0.929 (1.123 - 1.272)	0.903	-	-
Secondary school vs illiterate	1.560 (0.730 - 1.619)	0.458	-	-
High school vs illiterate	1.950 (0.335 - 1.671)	0.192	0.320 (0.105 - 0.970)	0.044
University vs illiterate	5.330 (0.834 - 2.513)	<0.001*	0.108 (0.038 - 0.301)	<0.001*
RA vs non-RA	1.73 (0.547 - 0.625)	0.896	-	-

Factors Associated With CIR Medication Discontinuation

In multivariate analysis, the factor associated with the discontinuation of CIR medication was a change in dietary habits (OR: 8.131; 95% CI (0.179 - 4.012); P = 0.032); the results are shown in Table 4.

**Table 4:** Univariate and multivariate analysis of CIR medication discontinuation *P-value significant at <0.05. P, probability of error

Parameters	Univariate analysis		Multivariate analysis	
	OR (CI 95%)	P-value	OR (CI 95%)	P-value
Age	0.044 (0.003 - 0.621)	0.879	-	-
Gender	2.616 (0.563 - 12.141)	0.219	-	-
Change in dietary habits	0.232 (0.044 - 1.231)	0.086	8.131 (0.179 - 4.012)	0.032*
Dietary supplements intake	1.662 (0.360 - 7.659)	0.514	-	-

## Discussion

Nutrition plays a crucial role in the management of chronic diseases, particularly CIR [7]. The data from our study reveal significant gaps in the nutritional knowledge of patients with CIR. While over half the participants acknowledged the importance of diet, many remained uncertain or misinformed about which foods to consume or avoid, particularly regarding dairy products and red meats. A substantial 40.5% of patients erroneously believed that red meat should be avoided, and similar percentages held misconceptions about dairy products. These beliefs contradict current SFR recommendations, which do not advocate for the exclusion of these food groups in CIR management [8,9].

In our study, around 40.5% of patients had erroneous beliefs about foods likely to aggravate the symptoms of CIR, including white flour, red meats, and dairy products. These results are in line with other studies, such as Tanner et al.'s study of 704 RA patients [10]. Another study investigating dietary beliefs in RA patients revealed results similar to ours: the majority of patients believed that eating fish improved their rheumatic symptoms, while sodas and red meats aggravated them [11]. Furthermore, a study involving 742 patients (290 with RA, 51 with juvenile RA, 87 with ankylosing spondylitis (AS), 51 with PsA, 65 with primary fibromyalgia, and 34 with osteoarthritis) demonstrated that 33% of RA patients reported worsening of their disease, joint pain and swelling, after consuming certain foods such as meat, alcohol, coffee, and sugar [12]. However, a recent survey including 168 patients, found that red meats, fish, and legumes were perceived as symptom triggers, while honey, garlic, and olive oil were considered beneficial by patients [13].

Our findings indicate that 51.5% of patients associated white flour with symptom aggravation. While some studies have suggested the potential benefits of gluten-free diets in RA, further research is needed to confirm these findings [14]. A French double-blind study, with a gluten-free diet in both arms and the addition of gluten-containing and non-gluten-containing products, is currently underway to investigate the effects of a gluten-free diet in spondyloarthritis, but no such trials have been conducted in RA [15].

Our data indicate that 38% of patients modified their dietary habits post-diagnosis, with the majority reducing their intake of fatty foods (52.7%), red meats (55.5%), sugar (51.4%), and dairy products (43.8%). Conversely, many increased consumption of perceived beneficial foods like vegetables (68.5%), fruit (65.1%), olive oil (71.2%), and fish (58.2%). These findings resonate with Salminen et al.'s study, which included 164 patients, where 51% of RA patients declared that they had modified their diet after diagnosis. The main changes reported were reduced consumption of animal fats, sugar, and red meat and increased consumption of fruit and vegetables [16].

A recent French multicenter survey further supports these observations. Among 448 patients (123 RA and 161 AS), 26% of patients were following or had followed at least one diet and 42% of patients indicated that at least one food or drink had an effect on pain. Green vegetables, fruit, and oily fish were perceived as beneficial, while cow's milk, white flour, and red meat were linked to symptom exacerbation [17].

The dietary practices observed in our study, such as exclusion diets and changes in eating habits, may have repercussions on therapeutic adherence. Approximately 27% of patients reported fasting to alleviate symptoms, and some even adjusted or discontinued medication due to dietary changes.

The implications of these findings for clinical management are considerable. It is essential that healthcare professionals integrate nutritional advice as part of CIR management. A multidisciplinary approach that includes dieticians and health educators could improve patients' understanding and help them make informed dietary choices, thus promoting better adherence to treatment [18].

The strengths of this study include the sample size, which enables robust data analysis. However, certain limitations must be taken into account. Selection bias could have influenced the results, as patients who participated in the study may have different levels of motivation or engagement compared to those who did not. In addition, the use of a non-validated questionnaire could affect the generalizability of the results.

To deepen our understanding of the impact of specific educational interventions on nutrition, it would be pertinent to suggest additional surveys. Such studies could explore the effectiveness of targeted educational nutrition programs, assessing their impact on patients' knowledge, attitudes, and practices, as well as on their adherence to treatment.

## Conclusions

This study reveals significant gaps in the nutritional knowledge of patients with CIR. Many patients harbor inaccurate beliefs about the impact of diet on their symptoms, underlining the urgent need for improved nutritional education. To optimize CIR management, it is imperative to develop specific dietary guidelines tailored to the Moroccan context and integrate evidence-based nutritional information into comprehensive therapeutic education programs. By addressing these knowledge gaps and empowering patients with accurate information, healthcare providers can significantly enhance disease management and improve patient outcomes.

## Appendices

List of foods and drinks

1.     White flour

2.     Whole wheat

3.     Barley

4.     Gluten

5.     Millet

6.     White rice

7.     Brown rice

8.     Cow's milk

9.     Butter

10.  Cheese

11.  Yogurt

12.  Whey

13.  Red meat

14.  Industrial chicken

15.  Free-range chicken

16.  Fish

17.  Farm eggs

18.  Free-range eggs

19.  Lentils

20.  Chickpeas

21.  Cabbage

22.  Tomatoes

23.  Eggplants

24.  Potatoes

25.  Green peppers

26.  Spinach

27.  Garlic

28.  Onions

29.  Fruits

30.  Salt

31.  Black pepper

32.  White pepper

33.  Chili pepper

34.  Cinnamon

35.  Ginger

36.  Saffron

37.  Turmeric

38.  Oregano

39.  Thyme

40.  Verbena

41.  Mint

42.  Wormwood

43.  Tap water

44.  Mineral water

45.  Alcohol

46.  Sparkling water

47.  Soda

48.  Green tea

49.  Tea bags

50.  Filtered coffee

51.  Capsule coffee

52.  Refined sugar

53.  Dark chocolate

54.  Milk chocolate

55.  Olive oil

56.  Vegetable oil

57.  Pure honey

58.  Canned goods

59.  Deli meats

60.  Seeds

61.  Celery

62.  Parsley

63.  Coriander

Questionnaire

1. Nutrition plays an important role in the management of chronic inflammatory rheumatism

Yes

No

I don't know

2. There is a link between diet and the activity of chronic inflammatory rheumatism

Yes

No

I don't know

3. Diet is important in managing diseases that accompany chronic inflammatory rheumatism

Yes

No

I don't know

4. It is recommended for overweight patients to lose weight to control the activity of chronic inflammatory rheumatism

Yes

No

I don't know

5. It is recommended for overweight patients to lose weight to improve diseases associated with chronic inflammatory rheumatism

Yes

No

I don't know

6. Dietary practices can replace medications for chronic inflammatory rheumatism

Yes

No

I don't know

7. Dietary practices should take into account the patient's age

Yes

No

I don't know

8. Dietary practices should take into account diseases other than chronic inflammatory rheumatism for which the patient is being followed

Yes

No

I don't know

9. Dietary practices should take into account the treatments for chronic inflammatory rheumatism that the patient is taking

Yes

No

I don't know

10. Patients with chronic inflammatory rheumatism should not consume dairy products

Yes

No

I don't know

11. Patients with chronic inflammatory rheumatism should not consume red meat

Yes

No

I don't know

12. Patients with chronic inflammatory rheumatism should eat fruits

Yes

No

I don't know

13. Patients with chronic inflammatory rheumatism should eat vegetables

Yes

No

I don't know

14. Patients with chronic inflammatory rheumatism should eat fish

Yes

No

I don't know

15. Patients with chronic inflammatory rheumatism should consume olive oil

Yes

No

I don't know

16. I think that consuming certain foods in particular would influence (improve or worsen) the symptoms of my chronic inflammatory rheumatism

Yes

No

I don't know

17. For each food listed above, please indicate whether it worsens, improves, has no effect on your

symptoms, or if you don't know:

White flour

Whole wheat

Barley

Gluten

Millet

White rice

Brown rice

Cow's milk

Butter

Cheese

Yogurt

Whey

Red meat

Industrial chicken

Free-range chicken

Fish

Farm eggs

Free-range eggs

Lentils

Chickpeas

Cabbage

Tomatoes

Eggplants

Potatoes

Green peppers

Spinach

Garlic

Onions

Fruits

Salt

Black pepper

White pepper

Chili pepper

Cinnamon

Ginger

Saffron

Turmeric

Oregano

Thyme

Verbena

Mint

Wormwood

Tap water

Mineral water

Alcohol

Sparkling water

Soda

Green tea

Tea bags

Filtered coffee

Capsule coffee

Refined sugar

Dark chocolate

Milk chocolate

Olive oil

Vegetable oil

Pure honey

Canned goods

Deli meats

Seeds

Celery

Parsley

Coriander

18. I think that fasting improves the symptoms of chronic inflammatory rheumatism

Yes

No

I don't know

19. I feel a difference in my health when I change my eating habits

Yes

No

If yes, please specify the differences you feel

20. I think a healthy diet can cure my rheumatism without the need for medication

Yes

No

I don't know

21. Since my rheumatism diagnosis, I have modified my eating habits

Yes

No

If yes, check the modifications you have made:

22. What is your most frequent cooking method?

Frying in oil

Pan-frying

Baking

Grilling

Steaming

Boiling

23. Do you follow a particular diet?

Yes

No

If yes, which diet do you follow?

24. Do you take dietary supplements?

Yes

No

If yes, which supplements do you take?

25. Do you practice fasting to improve the activity of your chronic inflammatory rheumatism?

Yes

No

If yes, how do you fast?

26. Have you changed your eating habits and stopped taking your chronic inflammatory rheumatism medications?

Yes

No

27. Have you changed your eating habits and decreased the dosage of your chronic inflammatory rheumatism medications?

Yes

No
